# Microarray-based analysis of renal complement components reveals a therapeutic target for lupus nephritis

**DOI:** 10.1186/s13075-021-02605-9

**Published:** 2021-08-25

**Authors:** Tao Liu, Mingyue Yang, Ying Xia, Chuan Jiang, Chenxu Li, Zhenyu Jiang, Xiaosong Wang

**Affiliations:** 1 Department of Rheumatology and Immunology, The First Hospital of Jilin University, Changchun, 130021 China; 2 Department of Translational Medicine, The First Hospital of Jilin University, Changchun, 130021 China

**Keywords:** Lupus nephritis, Complement C3, TGFβ1, SB431542, C3aR1

## Abstract

**Background:**

Screening abnormal pathways and complement components in the kidneys of patients with lupus nephritis (LN) and NZB/W mice may help to identify complement-related therapeutic targets for LN.

**Methods:**

KEGG and GO enrichment assays were used to analyze kidney microarray data of LN patients and NZB/W mice. Immunohistochemistry and immunofluorescence assays were used to measure renal expression of complement-related proteins and TGFβ1. Cytokines were measured using RT-qPCR and ELISA.

**Results:**

We screened the renal pathogenic pathways present in LN patients and NZB/W mice and selected the complement activation pathway for further study. The results indicated greater renal expression of C1qa, C1qb, C3, C3aR1, and C5aR1 at the mRNA and protein levels. C3 appeared to be a key factor in LN and the renal signaling downstream of C1 was inhibited. There were significant correlations between the expression of TGFβ1 and C3. Analysis of primary cell cultures indicated that TGFβ1 promoted the expression of C3 and that a TGFβ1 antagonist decreased the levels of C3 and C3aR. TGFβ1 inhibition significantly inhibited the deposition of complement-related factors in the kidneys of NZB/W mice.

**Conclusions:**

At the onset of LN, there are significant increases in the renal levels of C3 and other complement pathway-related factors in patients with LN and NZB/W mice. C3 may lead to albuminuria and participate in the pathogenesis of LN. TGFβ1 promotes C3 synthesis, and TGFβ1 inhibition may block the progression of LN by inhibiting the synthesis of C3 and other complement components.

**Supplementary Information:**

The online version contains supplementary material available at 10.1186/s13075-021-02605-9.

## Background

Lupus nephritis (LN) is one of the most common and serious complications of systemic lupus erythematosus (SLE) [1]. A systematic review reported that 40 to 82% of Asian patients with SLE had LN [2]. LN is a major cause of renal failure in SLE patients, and it directly increases morbidity and mortality [3]. In-depth investigation of the molecular pathogenesis of LN may provide a foundation for more precise therapies. Given the limited availability of sequential biopsies from patients, it remains essential to study informative mouse models of LN. Cross-species analyses can identify genes or pathways that are relevant to human disease and can be further studied in mouse models. Therefore, we initially screened the pathogenic pathways in the kidneys of LN patients and NZB/W mice (a model of LN), and then selected common complement activation pathway for further study.

The in situ deposition of immune complexes from the circulatory system or kidney may promote the accumulation of inflammatory cells and cause kidney damage [4, 5]. Previous studies reported that complement components are produced by the liver, kidneys, brain, blood vessels, and other organs [6, 7]. Renal tubules and podocytes directly produce most of the C3 [8, 9], which is then cleaved into C3a and C3b. Cleavage of C3b by factor I results in the formation of C3c and C3d [10]. C3 plays an important role in the classical, lectin-mediated, and alternative immune pathways [11]. C3a is an allergic toxin that stimulates mast cell degranulation after binding to its receptor C3aR, and it also has chemotactic and antimicrobial activities in inflammatory responses [12].

Immunofluorescence studies indicated abundant deposition of TGFβ and fibrin in the renal tissues of patients with LN [13]. Our previous research found that TGFβ1 promoted the formation of platelet-derived growth factor subunit B (PDGF-B) [14]. Studies of patients with glomerulonephritis indicated that TGFβ1 and PDGF-B functioned as important mediators of extracellular matrix (ECM) accumulation, glomerular fibrosis, and mesangial cell proliferation [15–19]. Other research reported that the interaction between TGFβ1 and different complement components exacerbated epithelial damage in pulmonary fibrosis [20]. However, the relationship of TGFβ1 with complement components during the pathogenesis of LN is unclear.

We examined changes in the kidneys of patients with LN and a mouse model of LN (NZB/W mice). We focused on excessive activation of complement pathways and upregulation of specific complement components such as C3 that occur during the pathogenesis of LN. We also examined the effect of TGFβ1 on the expression of C3 at the mRNA and protein levels, and the effect of a TGFβ1 inhibitor (SB431542) on the synthesis of C3 and other complement components.

## Methods

### Patient data and samples

Complete clinical data were from SLE patients who were newly diagnosed from July 2018 to December 2019 at the First Hospital of Jilin University (Additional file 1: Supplementary Table S1). All patients had at least 4 of the 11 diagnostic criteria for SLE from the revised criteria of the American College of Rheumatology (ACR) and the American Rheumatology Association [21]. Among these patients with SLE, LN was defined as 24 h urinary protein of more than 0.5 g/day or 3+ on a dipstick test. There were 69 cases of SLE without LN and 76 cases of LN. Blood samples were collected before onset of treatment. Blood samples from 47 healthy persons (control) who were matched for age and sex were also collected. Peripheral blood mononuclear cells (PMBCs) were isolated from whole blood by density gradient centrifugation.

The renal tissues of 23 patients with LN were collected and classified according to the International Society of Nephrology/Renal Pathology (ISN/RPS) 2003 criteria [22]. Renal tissues were also collected from 13 controls; these control tissues were from healthy living donors before kidney transplantation (*n* = 6) or normal kidney tissues distant from the tumors of patients with cancer (*n* = 7).

### Ethical approval and consent to participate

Ethical approval for this study was received from the Institutional Medical Ethics Review Board of the First Hospital of Jilin University (reference number: 2017-087). All procedures were in compliance with the Declaration of Helsinki.

### Microarray analysis

Microarray analysis of gene expression profiles was based on data from the GEO database [23]. Kidney tissues were from the whole kidneys of NZB/W mice (accession number: GSE32583) or human glomerular and renal tubular tissues (accession number: GSE32591). Mice were divided into three groups: control (16-week-old NZB/W mice without disease, *n* = 8); LN1 (23-week-old NZB/W mice with proteinuria and glomerulonephritis, *n* = 6); and LN2 (36-week-old NZB/W with proteinuria and glomerulonephritis, *n* = 10). Details are provided at the GEO (https://www.ncbi.nlm.nih.gov/geo/geo2r/?acc=GSE32583). Samples of human glomeruli and renal tubules were from renal biopsies [23]. The glomerular samples were divided into two groups: Ctrl-GLO (glomeruli from control living donors, *n* = 14) and LN-GLO (glomeruli from LN patients, *n* = 32). Renal tubule samples were divided into two groups: Ctrl-Tub (tubulointerstitium from control living donors, *n* = 15) and LN-Tub (tubulointerstitium from LN patients, *n* = 32). Details are provided at the GEO (https://www.ncbi.nlm.nih.gov/geo/geo2r/?acc=GSE32591). The standard of a *p* value below 0.05 for a fold-change in expression of more than 1.2 was used for differential gene analysis.

### Immunohistochemistry and Immunofluorescence staining

An immunohistochemistry assay was used to examine kidney tissues that were fixed in 10% neutral formalin solution, embedded in paraffin, and then dewaxed and sliced. After incubation at room temperature with an oxidase blocking solution, animal serum was added, and then a primary antibody against different proteins (C3, C3aR, C5, C5aR1, C1q, or TGFβ1; Bioss Biotechnology Co. LTD, Beijing, China) was added for overnight incubation. Then the secondary antibody and hematoxylin were added for visualization. The cell staining score was determined using Image J software [24]. The positive ratio of positive cells to total cells was determined by an average of five readings of each sample.

An immunofluorescence assay was used to examine kidney tissues embedded in optimal cutting temperature compound (OCT), frozen, and sectioned. After drying at room temperature, sections were stained with Complement C3 Antibody 11H9 (Cat. # NB200-540AF594, Novus Biologicals, shanghai, China, Alexa Fluor 594). Sections were then incubated in darkness with 11H9 (1.1 mg/mL, 1:200) for 1 h at 37 °C. After washing and drying, 50 μL of Hoechst 33342 (5 mg/mL, Sigma-Aldrich, Germany) was added, and the sample was incubated in darkness for 20 min at 37 °C for nuclear staining. The sections were then sealed and observed using laser confocal microscopy.

### ELISA

A human complement protein C3 ELISA kit (#CSB-E08665h, Wuhan Huamei, China) was used to measure C3 according to the manufacturer’s instructions. A human TGFβ1 ELISA kit was used to measure TGFβ1 in plasma using a previously described procedure [14]. A human TGFβ1 ELISA kit (#EK0513, Boster, China) was used to measure TGFβ1 in urine following the manufacturer’s instructions.

### Real-time quantitative PCR

Cells were treated with the TRIzol reagent (Invitrogen, Carlsbad, California, USA), stored at – 80 °C (5 × 10^6^ cells/mL) for extraction of total RNA, and RT-qPCR was then performed as previously described [14].

### Animal experiments

Female NZB/W mice were purchased from Jackson Laboratory (Bar Harbor, USA). Mice were randomly divided into two groups at the age of 16 weeks. One group received 4 cycles of 200 μL (1.5 mg/mL) intraperitoneal injections of SB431542 and the other group received the same routine with saline. Each treatment cycle consisted of an injection every other day for 1 week, followed by no injections for 3 weeks. The mice were sacrificed at 32 weeks of age, and the kidneys were then harvested for analysis.

### Cell culture

Mouse bone marrow cells were extracted under sterile conditions, and erythrocytes were lysed. Mouse bone marrow cells were cultured using DMEM with penicillin (100 U/mL), streptomycin (100 μg/mL), and 10% FBS. These cells were divided into three groups: control, mouse recombinant TGFβ1 (2.5 ng/mL R&D Systems, Minneapolis, MN, USA), and SB431542 (5 μM; Sigma-Aldrich, St. Louis, MO, USA).

Mouse fresh kidney samples were fully shredded to a size of 1 mm^3^ using ophthalmic scissors. A type I collagenase solution (0.1 U/mL) was added, followed by shaking for 60 min at 37 °C. Single primary renal cells were obtained by filtration and cultured in RPMI1640 with medium penicillin (100 U/mL), streptomycin (100 μg/mL), and 10% FBS. The cells were divided into three groups: control, mouse recombinant TGFβ1 (2.5 ng/mL), and SB431542 (5 μM).

Human PBMCs were cultured in RPMI 1640 medium with penicillin (100 U/mL), streptomycin (100 μg/mL), and patient plasma (9:1 ratio of RPMI 1640: plasma). The cultured cells were divided into three groups: control; human recombinant TGFβ1 (7.5 ng/mL, R&D Systems, Minneapolis, MN, USA); and SB431542 (5 μM).

All cells were collected after culturing in an incubator (37 °C and 5% CO_2_) for 5 h.

### Statistical methods

GraphPad Prism version 8.0 (San Diego CA, USA) was used for statistical analysis and production of figures. To compare different matrices, all data between the maximum (red) and minimum (blue) values for each gene expression level were used to generate an unsupervised heat map. The Wilcoxon signed-rank test was used to compare paired samples, the Mann-Whitney U test was used to compare unpaired samples, and the nonparametric Spearman rank correlation test was used to determine correlations. A *p* value below 0.05 was considered significant.

## Results

### Activation of complement pathways and upregulation of complement components in kidneys of patients with LN

Our initial microarray analysis compared renal tissue samples from healthy controls and patients with LN and identified the presence of 4754 differentially expressed genes, with 2632 upregulated genes and 2122 downregulated genes in the glomeruli of LN patients. (Additional file 2: Supplementary Figure S1). A similar analysis indicated there were 3725 differentially expressed genes in the renal tubules, with 2359 upregulated genes and 1366 downregulated genes in the renal tubules of LN patients (Additional file 2: Supplementary Figure S1).

**Fig. 1:**
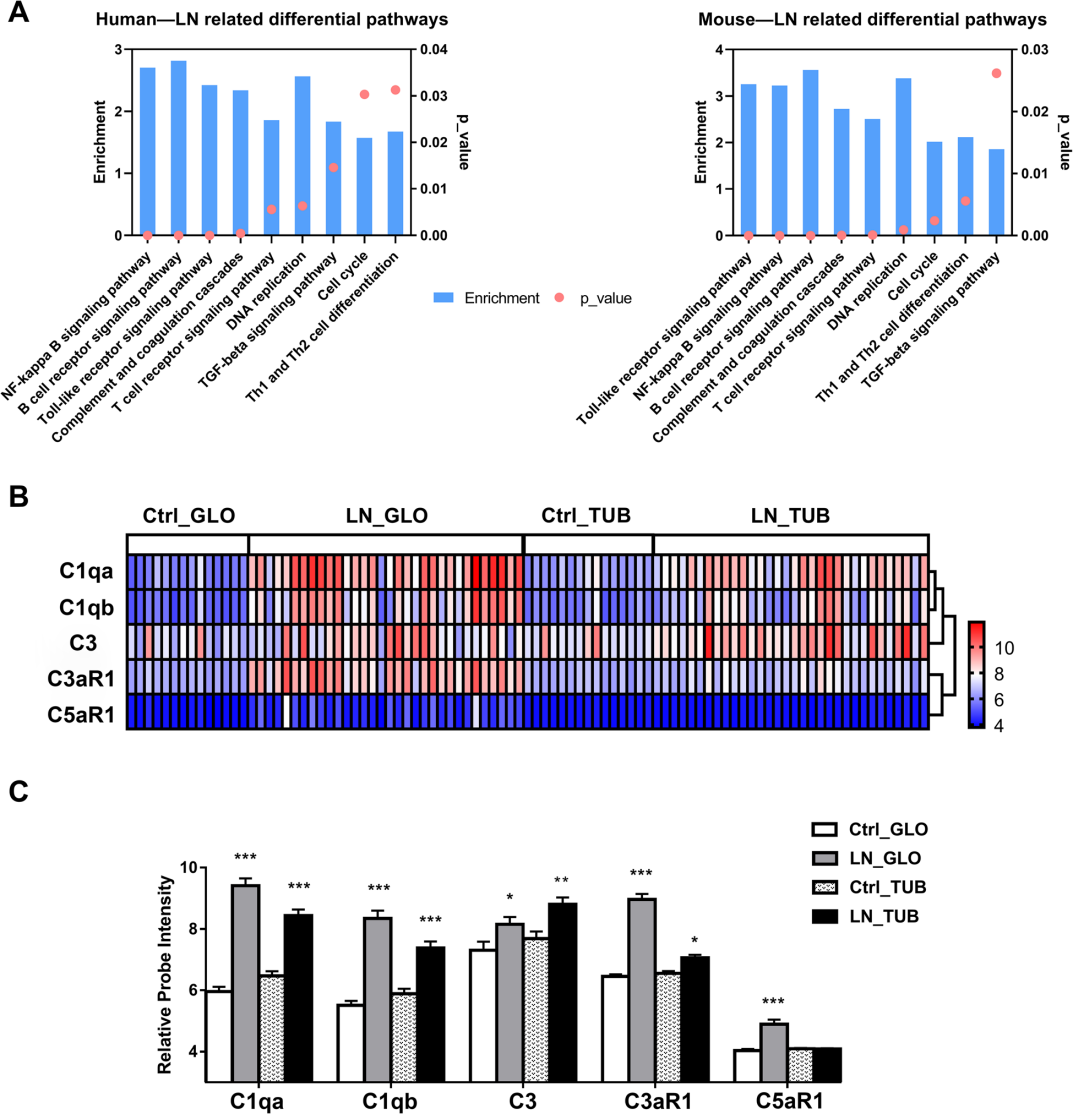
Renal pathways and complement genes in LN patients and healthy controls based on microarray results. **A** Enrichment and p-values of some upregulated KEGG pathways in the kidneys of LN patients and NZB/W F1 mice. **B** Cluster analysis of the expression of C1qa, C1qb, C3, C3aR1, and C5aR1 in the glomeruli (GLO) and renal tubules (TUB) in each group. **C** mRNA levels of C1qa, C1qb, C3, C3aR1, and C5aR1 in the renal tubules of patients with LN (*N* = 32) and healthy controls (*N* = 15). * *p* < 0.05, ** *p* < 0.01, *** *p* < 0.001

KEGG enrichment analysis indicated there were 89 significantly upregulated pathways in LN patients (Additional file 3: Supplementary Figure S2). We also compared the differentially regulated pathways in LN patients and model mice, to identify pathways that were altered in both (Fig. 1A). These pathways included NF-kappa B signaling pathway, B cell receptor signaling pathway, Toll-like receptor signaling pathway, complement and coagulation cascades, T cell receptor signaling pathway, TGFβ signaling pathway, and several other pathways (Additional file 4: Supplementary Table S2).

Cluster analysis showed close similarities in the expression of C1qa with C1qb, of C3aR1 with C5aR1, and of C3 with C1qa and C1qb (Fig. 1A). Quantitation of these microarray data (Fig. 1B) indicated that LN patients had increased expression of C1qa, C1qb, C3, C3aR1, and C5aR1 in glomeruli, and increased expression of all these mRNAs except C5aR1 in renal tubules.

**Fig. 2:**
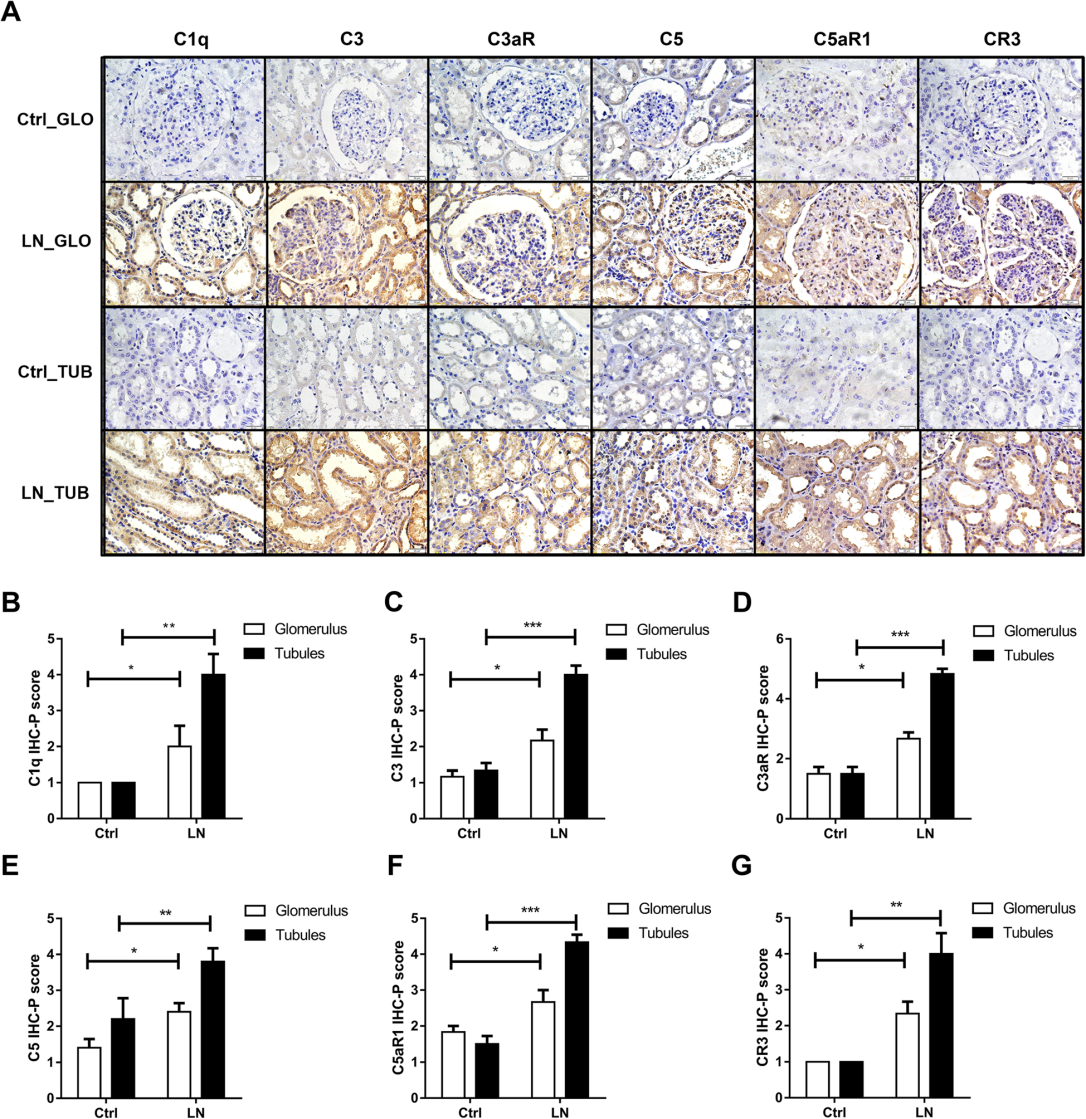
Immunohistochemical analysis of complement components in the glomeruli and renal tubules of LN patients and healthy controls. **A** Expression of C1q, C3, C5, C3aR, C5aR1, and CR3 in glomeruli (GLO) and renal tubules (TUB). **B** Immunohistochemistry -paraffin section (IHC-P) scores of C1q (*N* = 3, *p* < 0.01). **C** IHC-P scores of C3 (*N* = 6, *p* < 0.001). **D** IHC-P scores of C3aR (*N* = 5, *p* < 0.01). **E** IHC-P scores of C5 (*N* = 6, *p* < 0.001). **F** IHC-P scores of C5aR1 (*N* = 6, *p* < 0.001). **G** The IHC-P scores of CR3 (*N* = 3, *p* < 0.01). All IHC-P scores were determined using ImageJ software

Next, we performed immunohistochemical analysis of the kidneys of LN patients and healthy controls. The results indicated greater protein levels of C1q, C3, C5, C3aR, C5aR1, and CR3 in the renal tubules of patients with LN (Fig. 2A). Quantitation of expression using cell staining scores from Image J software confirmed that these results were significantly different in the renal tubules and glomeruli (Fig. 2B–G). These results suggested that the increased protein levels in the kidneys of patients with LN may be related to over-activation of complement pathways.

### Activation of complement pathways and upregulation of complement components in kidneys of NZB/W mice

We further analyzed the kidneys of NZB/W mice. A comparison of whole kidney tissues before and at early-stage disease indicated 1725 differentially expressed genes, with 1375 upregulated genes and 350 downregulated genes in mice with early-stage disease (Additional file 2: Supplementary Figure S1). A comparison of whole kidney tissues before and at late-stage disease indicated 7966 differentially expressed genes, with 2863 upregulated genes and 5103 downregulated genes in the mice with late-stage disease (Additional file 2: Supplementary Figure S1).

**Fig. 3:**
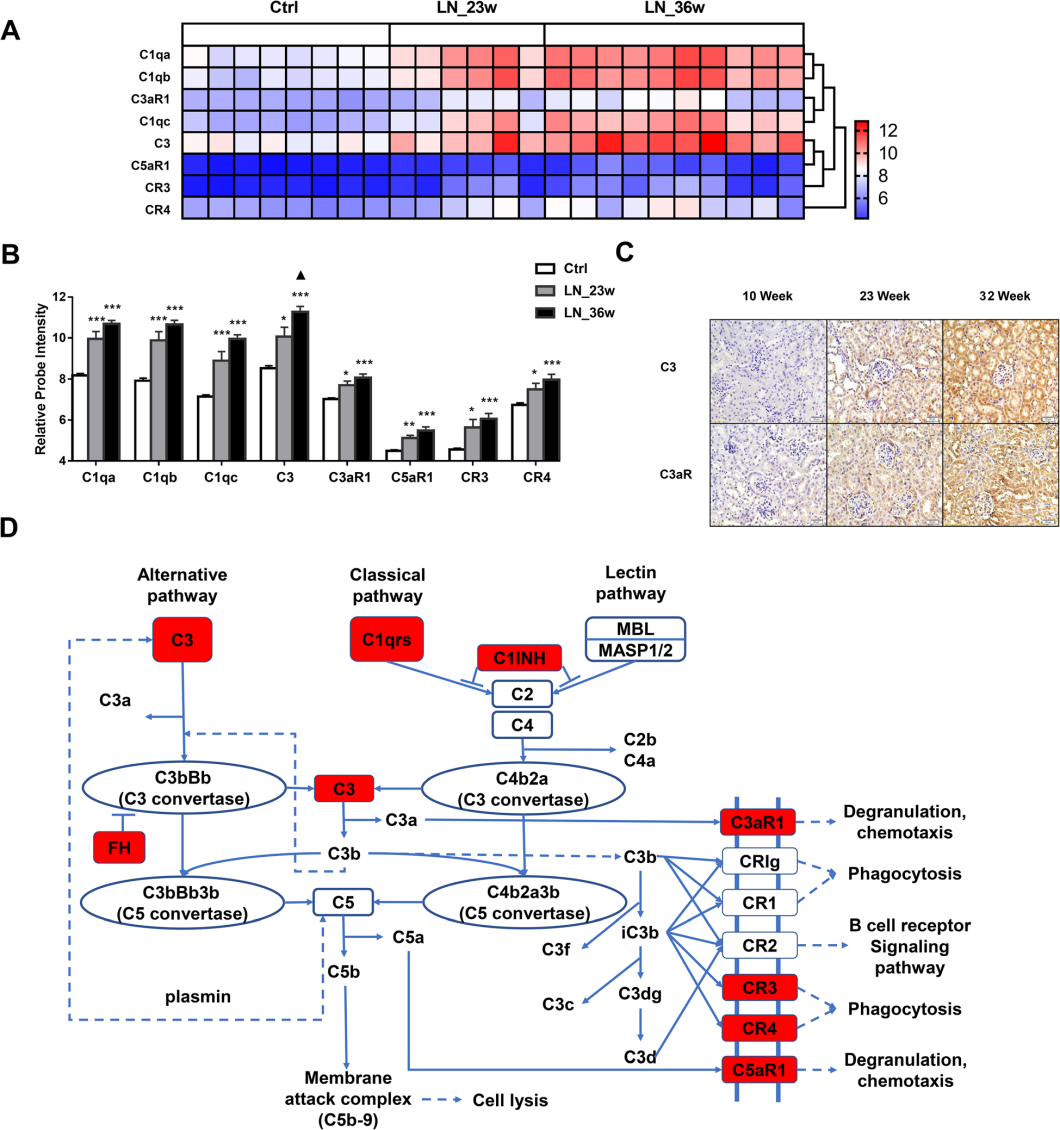
Expression of renal complement components in NZB/W mice during the progression of LN. **A** Cluster analysis showing the expression of C1qa, C1qb, C1qc, C3aR1, C3, C5aR1, CR3, and CR4 in the control group (16 weeks, *N* = 8), early-stage group (23 weeks, *N* = 6), and late-stage group (36 weeks, *N* = 10). **B** Renal mRNA levels of complement components in each group (* *p* < 0.05, ** *p* < 0.01, *** *p* < 0.001, ▲ significant difference between late-stage and early-stage). **C** Immunohistochemical analysis of C3 and C3aR. **D** Complement activation pathway analysis based on kidney microarray results of LN patients and NZB/W mice, red boxes show significantly upregulated genes

Cluster analysis of complement-related factors in these mice indicated that the expression of C1qa, C1qb, C1qc, C3, C3aR1, C5aR1, CR3, and CR4 in NZB/W mice gradually increased during disease progression (Fig. 3A). These changes had some similarities to those in human glomeruli and renal tubules. In particular, the expression of C1qa and C1qb clustered together (as in humans), the expression of C1qc and C3aR1 clustered together. However, the expression of C3 clustered with C5aR1, and the expression pattern of C3aR1 was similar to C1qa, C1qb, and C1qc.

Quantitation of the microarray data (Fig. 3B) indicated progressively increased expression of C1qa, C1qb, C1qc, C3, C3aR1, C5aR1, CR3, and CR4 as the disease progressed. In particular, C3 mRNA had significantly greater expression at late-stage disease than early-stage disease.

We also performed immunohistochemical analysis of C3 and C3aR in the kidneys of five NZB/W mice. The results indicated increasing deposition of both proteins as the disease progressed (Fig. 3C). These results confirmed that changes in complement components were associated with the pathogenesis of LN in this mouse model.

To identify the upstream and downstream relationships of these upregulated genes (C1q, C3, C3aR1, C5aR1, CR3, and CR4), we performed KEGG pathway analysis using the lupus mouse microarray database (Fig. 3D). The starting component C1qrs activates the downstream complement C3, which is then cleaved into two downstream factors—C3a and C3b. The binding of different complements to their receptors (C3aR1, C5aR1, CR3, etc.), induces responses, including phagocytosis, degranulation, and chemotaxis. We found significant upregulation of C1q, C3, CR3, CR4, C3aR1, and C5aR1. We also found upregulation of C1INH (also known as SERPING1), which is known to inhibit downstream signaling of the classical and lectin pathways. These results confirmed changes in the levels of different complement components function in the pathogenesis of LN.

### Complement related upregulated GO terms and C3 levels in LN patients

**Fig. 4:**
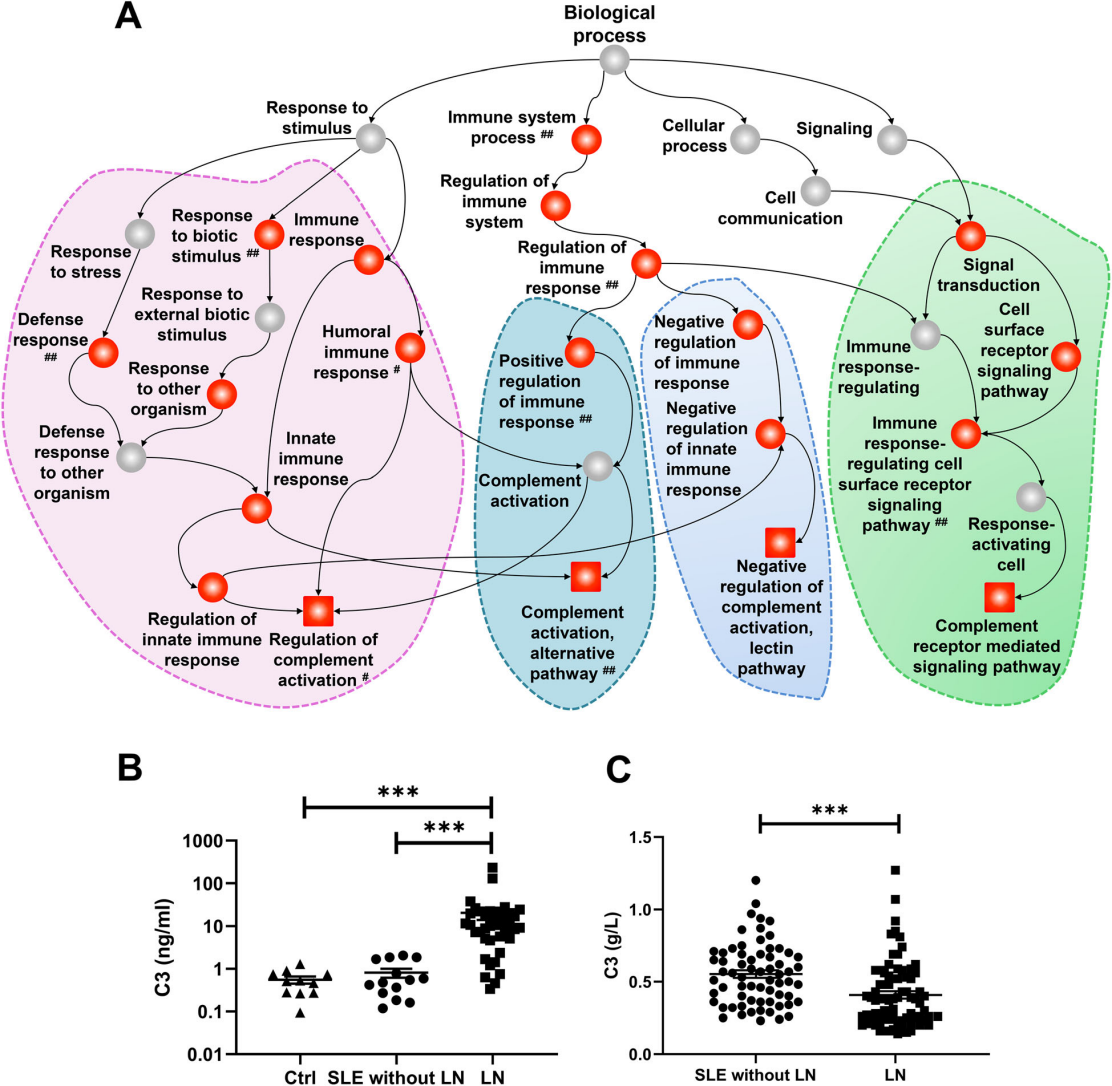
GO enrichment analysis and the levels of complement C3 in LN patients. **A** Directed acyclic graph of GO enrichment, showing upregulated genes in biological process in LN patients and NZB/W mice. Squares: complement-related GO terms; red squares or red circles: significant enrichment; # significant enrichment only in LN patients; ## significant enrichment only in NZB/W mice; the unmarked terms: have significant enrichment in LN patients and NZB/W mice. **B** Levels of urinary C3 in healthy controls (*N* = 11), SLE patients without LN (*N* = 14) and patients with LN (*N* = 39). *** *p* < 0.001. **C** Levels of plasma C3 in SLE patients without LN (*N* = 66) and patients with LN (*N* = 76). *** *p* < 0.001. **D** Correlation between the urinary levels of C3 and 24 h urine protein (*N* = 37)

GO enrichment analysis of biological process class help us to explore the biological functions of the differentially expressed genes. We chose the immune response related complement and coagulation cascades pathway for further study. The complement related upregulated genes were depicted as directed acyclic graphs in Fig. 4A. The detailed information of the significantly increased complement-related GO terms was listed in Table 1. The results indicated significantly upregulated genes, including serpin family G member 1 (SERPING1) and alpha-2-macroglobulin (A2M), which were significantly enriched in the GO term of negative regulation of complement activation, lectin pathway (GO: 0001869) in the kidneys of LN patients and NZB/W mice. This result suggests inhibition of downstream signaling of C1 in the kidneys of LN patients. The significantly upregulated genes including formyl peptide receptor 1 (FPR1), formyl peptide receptor 2 (FPR2), and complement C3a receptor 1 (C3aR1) were significantly enriched in the GO term of complement receptor-mediated signaling pathway (GO: 0002430) in the kidneys of LN patients and NZB/W mice. This result suggests that C3a may be a key pathogenic factor in LN patients. Upregulation of the term of regulation of complement activation (GO: 0030449) was significant only in LN patients, and upregulation of the term of complement activation, alternative pathway (GO: 0006957) was significant only in NZB/W mice.

**Table 1:** Details of the four significantly different GO terms associated with complement

Species	GO_ID	GO_Name	Population_mapped_id	Study_mapped_id	Gene_symbol	Description	Enrichment	***p*** value	FDR
Human	GO:0001869	Negative regulation of complement activation, lectin pathway	2	2	SERPING1, A2M	Serpin family G member 1, alpha-2-macroglobulin	14.158	0.005	0.062
Mouse	GO:0001869	Negative regulation of complement activation, lectin pathway	2	2	Serping1, A2m	Serine (or cysteine) peptidase inhibitor, clade G, member 1, alpha-2-macroglobulin	21.701	0.002	0.019
Human	GO:0002430	Complement receptor-mediated signaling pathway	9	3	FPR1, FPR2, C3AR1	Formyl peptide receptor 1, formyl peptide receptor 2, complement C3a receptor 1	4.719	0.021	0.153
Mouse	GO:0002430	Complement receptor-mediated signaling pathway	11	4	Fpr1, C3ar1, Fpr2, C5ar1	Formyl peptide receptor 1, complement component 3a receptor 1, formyl peptide receptor 2, complement component 5a receptor 1	7.891	0.001	0.013
Human	GO:0030449	Regulation of complement activation	29	7	C5AR1, C3, C3AR1, C1QBP, CFH, CFP, PROS1	Complement C5a receptor 1, complement C3, complement C3a receptor 1, complement C1q binding protein, complement factor H, complement factor properdin, protein S (alpha)	3.417	0.003	0.052
Mouse	GO:0006957	Complement activation, alternative pathway	10	3	C3, Cfh, Cfp	Complement component 3, complement component factor h, complement factor properdin	6.510	0.009	0.055

To further examine the possible role of C3 in the pathogenesis of LN, we examined the expression of C3 in the urine, blood, and kidney tissues of LN patients. The results indicated the LN group had a significantly higher urinary level of C3 than the healthy controls and the SLE without LN group (both *p* < 0.001; Fig. 4B). However, the plasma of the LN group had a decreased level of C3 (*p* < 0.001; Fig. 4C) relative to the SLE without the LN group. This suggests that C3 was excreted from the kidneys of patients with LN. The plasma of the LN group also had a lower level of C1q than the SLE without the LN group (*p* < 0.05; Additional file 5: Supplementary Figure S3A), but these groups had no significant difference in the level of C4 (p > 0.05; Additional file 5: Supplementary Figure S3B). We also observed significant correlations of the plasma levels of C3 with C1q (*p* < 0.0001, *r* = 0.6345; Figure S3 C) and with C4 (*p* < 0.0001, *r* = 0.5261; Figure S3D). These results suggest that an elevated level of C3 may contribute to the pathogenesis of LN and proteinuria. Our immunohistochemistry analysis indicated that the expression of C3 was similar in LN patients with class IV, V, and IV+V disease, but the expression was greater in those with class III disease than healthy controls (Additional file 6: Supplementary Figure S4).

### Upregulation of TGFβ1 and C3 during pathogenesis of LN

**Fig. 5:**
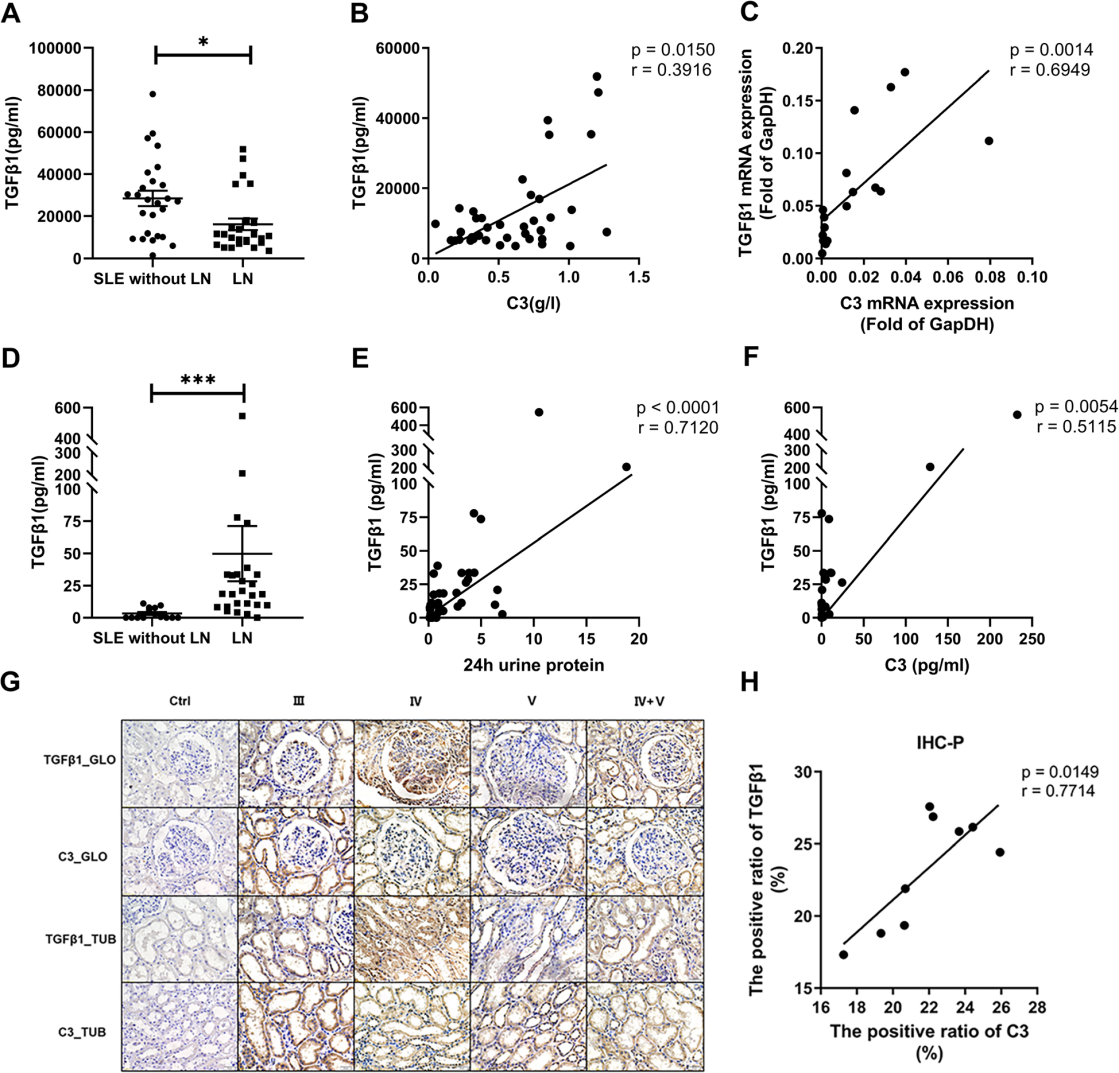
Relationships between the levels of TGFβ1 and C3 in LN patients. **A** Plasma levels of TGFβ1 in SLE patients without LN (*N* = 26) and patients with LN (*N* = 25). * *p* < 0.05. **B** Correlation of the protein levels of TGFβ1 and C3 in plasma of LN patients (*N* = 38). **C** Correlation of the mRNA levels of TGF β1 and C3 in PBMCs of LN patients (*N* = 18). **D** Urine levels of TGFβ1 in SLE patients without LN (*N* = 14) and patients with LN (*N* = 26). *** *p* < 0.001. **E** Correlation between the levels of TGFβ1 and 24 h urine protein in patients with LN (*N* = 26). **F** Correlation between the urine levels of TGFβ1 and C3 in LN patients (*N* = 26). **G** Immunohistochemical analysis of TGFβ1 and C3 in the glomeruli (GLO) and renal tubules (TUB) of patients with different classes of LN

We previously reported a correlation between the levels of TGFβ1 and C3 in whole blood cells of SLE patients [25] and proposed that the levels of TGFβ1 and C3 may have similar changes in the urine and blood of patients with LN. Our analysis of plasma in the LN group indicated a decreased level of TGFβ1 relative to the SLE without the LN group (*p* < 0.05; Fig. 5A). Our measurements of TGFβ1 and C3 in patients with LN indicated positive correlations in the levels of these proteins (*p* = 0.0150, *r* = 0.3916; Fig. 5B) and mRNAs (*p* = 0.0014, *r* = 0.6949; Fig. 5C) in blood. However, the LN group had a significantly higher urinary level of TGFβ1 than the SLE without the LN group (*p* < 0.001; Fig. 5D). Analysis of the relationship between urinary TGFβ1 and 24 h urinary protein indicated a significantly positive correlation (*p* < 0.0001, *r* = 0.7120; Fig. 5E). In addition, the levels of TGFβ1 were significantly correlated with that of C3 in the urine of these patients (*p* = 0.0054, *r* = 0.5115; Fig. 5F). Our renal immunohistochemistry results (Fig. 5G) also indicated a correlation of cell staining scores for TGFβ1 and C3 (*p* = 0.0149, *r* = 0.7714; Fig. 5H). These findings suggest that TGFβ1 and C3 function in the pathogenesis of LN.

### TGFβ1 upregulates C3

**Fig. 6:**
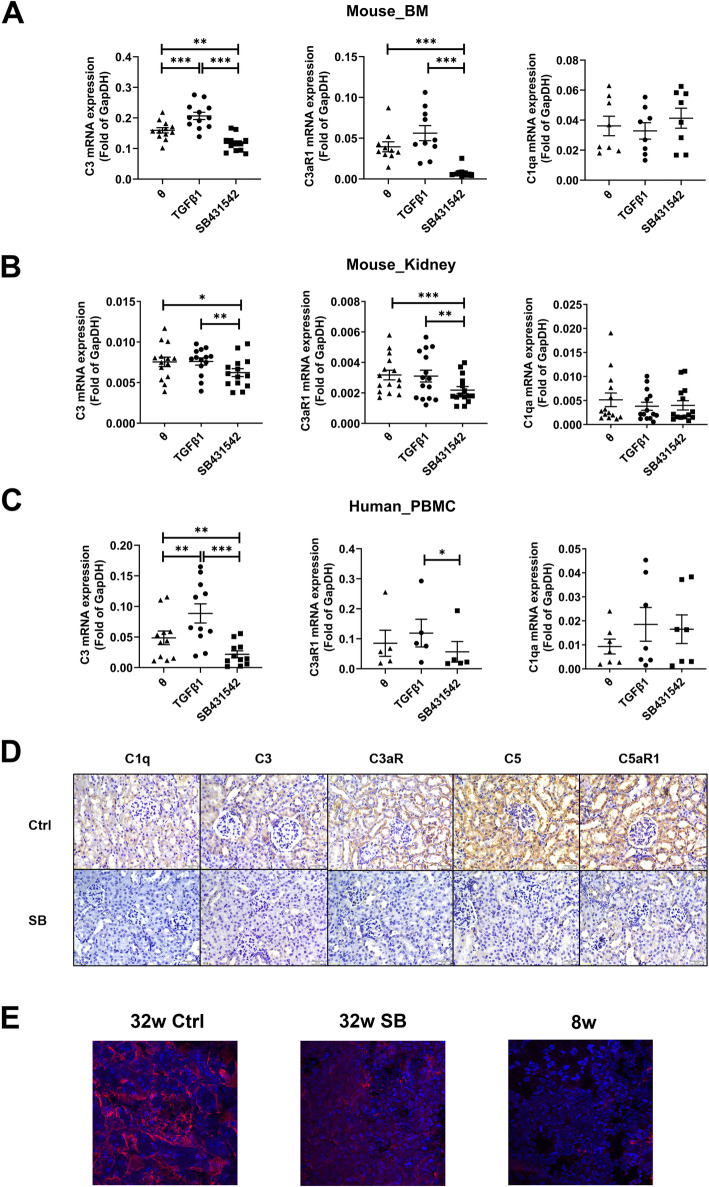
Effects of TGFβ1 on the production of C3 and other complement components. **A** mRNA levels of C3 (*N* = 12), C3AR1 (*N* = 10), and C1qa (*N* = 8) in the presence of TGFβ1 or SB431542 in bone marrow cell-cultures of NZB/W mice. **B** mRNA levels of C3 (*N* = 14), C3aR1 (*N* = 15), and C1qa (*N* = 14) in the presence of TGFβ1 or SB431542 in kidney primary cell-cultures of NZB/W mice. **C** mRNA levels of C3 (*N* = 11), C3AR1 (*N* = 5), and C1qa (*N* = 7) in the presence of TGFβ1 or SB431542 in PBMC-cultures of LN patients. * *p* < 0.05, ** *p* < 0.01, *** *p* < 0.001. **D** Immunohistochemical analysis of C1q, C3, C5, and their receptors (C3aR, C5aR1) in the kidneys of NZB/W mice treated with SB431542 or saline as control. **E** Immunofluorescence analysis of C3 in the kidney of NZB/W mice: 32-week-old mice treated with SB431542, 32-week-old mice treated with saline, and 8-week-old mice without treatment. Red fluorescence indicates C3 and blue fluorescence indicates nuclei

To confirm that TGFβ1 regulates the expression of C3, we cultured NZB/W mouse bone marrow cells with TGFβ1 or SB431542. The results indicated that TGFβ1 significantly increased the expression of C3 mRNA (*p* < 0.001; Fig. 6A) and that SB431542 significantly decreased the expression of this mRNA (*p* < 0.01; Fig. 6A). Interestingly, SB431542 also decreased the level of C3aR1 mRNA (*p* < 0.001; Fig. 6A), although TGFβ1 had no significant effect on this mRNA (*p* > 0.05; Fig. 6A). TGFβ1 and SB431542 had no significant effects on the level of C1q mRNA (*p* > 0.05; Fig. 6A).

We used the same method to culture NZB/W mouse kidney primary cells. The results showed that SB431542 decreased the mRNA levels of C3 (*p* < 0.05; Fig. 6B) and C3aR1 (*p* < 0.001; Fig. 6B), although TGFβ1 had no significant effect on these mRNAs (both *p* > 0.05; Fig. 6B). TGFβ1 and SB431542 also had no significant effect on C1q mRNA (*p* > 0.05; Fig. 6B).

We performed similar experiments using the PBMCs from SLE patients. TGFβ1 and SB431542 had the same effects on C3 mRNA (both *p* < 0.01; Fig. 6C). However, analysis of C3aR1 indicated that TGFβ1 and SB431542 had no significant effect relative to the controls, although expression of C3aR1 mRNA was greater in cells given TGFβ1 than SB431542 (*p* < 0.05; Fig. 6C). TGFβ1 and SB431542 had no significant effect on C1q mRNA (both *p* > 0.05; Fig. 6C).

We also tested the effect of 3 months of administration of SB431542 or saline on NZB/W mice. The results indicated that SB431542 significantly reduced the levels of C1q, C3, C5, C3aR, C5aR1, and CR3 (Fig. 6D). To confirm these results, we performed immunofluorescence staining of C3 using a different antibody. These results also indicated that SB431542 significantly reduced the levels of C3 (Fig. 6E).

## Discussion

We screened and analyzed the kidney genomic data of LN patients and NZB/W mice, to identify pathways related to the pathogenesis of this disease, with a focus on complement activation. Our results indicated the activation of various complement-related factors and GO terms, and subsequent analysis suggested they had roles in the pathogenesis of LN. In particular, the results suggested that C3 was a key factor in LN and the signaling downstream of C1q was inhibited in the kidneys of patients with this disease. TGFβ1 was associated with an increased level of C3 in NZB/W mice and LN patients, but was unrelated to C1q. A TGFβ1 inhibitor (SB431542) significantly inhibited C3 synthesis in primary kidney cells and peripheral blood cells and reduced the complement deposition in the kidneys of NZB/W mice.

To provide foundation for the more effective treatment of LN, we identified pathways that had differential expression in LN patients and model mice, relative to controls. We also used bioinformatics to analyze mRNA expression of complement-related factors in the kidney tissues of LN patients and NZB/W mice. KEGG enrichment analysis showed similarities in differential expression pathways of complement and coagulation cascades in LN patients and NZB/W mice. The present study is the first to use big data from microarray analysis to demonstrate that the mRNA levels of multiple complements (C1qa, C1qb, C3) and complement receptors (C3aR1 and C5aR1) were upregulated in the glomeruli and renal tubules of patients with LN and the results were generally similar in the kidney tissues of NZB/W mice. Moreover, as the disease progressed in these mice, the levels of C3 and C1q (C1qa, C1qb, C1qc), C3, C3aR, C5aR1, CR3, and CR4 continued to increase. We confirmed the deposition of complement-related factors including C1q, C3, C3aR, C5, and CR3 by immunohistochemistry in the kidneys of LN patients. Further analysis of mice indicated significant upregulation of C1q mRNA (an upstream component) as well as C3 and its receptors C3aR1, CR3, and CR4 (downstream components). Therefore, there is upregulation of numerous complement-related factors and pathways in the kidneys of LN patients.

Previous studies on mouse models of lupus (NZB/W and MRL/*lpr* mice) reported increased renal expression of complement mRNAs and proteins [26, 27]. Moreover, complement inhibitors reduced proteinuria and prevented the deterioration of renal function in these animal models [26, 27]. Excessive activation of the renal complement system can cause renal tubular interstitial cytotoxicity, in which renal tubular epithelial cells are a key target of C3, resulting in proteinuria and promotion of renal fibrosis [28–30]. These previous results are consistent with our results, and taken together confirm the presence of complement pathway activation in the kidneys of patients with LN.

Our GO enrichment analysis showed the presence of an activated immune response cascade. The upregulated FPR1, FPR2, and C3aR1 were significantly enriched in the GO term of complement receptor-mediated signaling pathway in the kidneys of LN patients and NZB/W mice. FPR1 and FPR2 are Gi-protein-coupled receptors that are expressed mainly by mammalian phagocytic leukocytes; FPRs are involved in antibacterial host defense and inflammation; C3aR1 is a receptor for the chemotactic and inflammatory peptide anaphylatoxin C3a. Our results suggest that C3 is a key pathogenic factor in the kidneys of LN patients. C3 functions as the core of the three pathways of complement system activation, and forms a C5b-9 membrane attack complex that attacks kidney tissues *via* a cascade reaction [31]. This leads to the release of proinflammatory mediators and other factors (e.g., reactive oxygen species and tissue degrading proteases) that damage the surfaces of endothelial cells and the glomerular basement membrane, destroy glomerular podocyte foot processes, and disrupt kidney filtration, ultimately leading to proteinuria, kidney tissue damage, and then LN [31].

We found that the reduced blood level of C3 in patients with LN was associated with an increased level of C3 in their urine. Liu et al. [32] reported that a lower serum level of C3 was associated with a higher risk of focal segmental glomerulosclerosis and end-stage renal disease. Our results also indicated that NZB/W mice had an increasing renal expression of C3 and C3aR as the disease progressed. This is consistent with previous reports on the deposition of large amounts of C3 in the kidneys of patients with LN and in mouse models of this disease [33, 34]. Our finding of a significant decrease of plasma C3 in the presence of LN is consistent with previous clinical and animal experiments which reported that complement activation leads to secondary decreases in the blood levels of C4 and C3 during the active period of SLE [35]. We speculate that the loss of C3 through urine and deposition in the kidneys were partly responsible for the decreased plasma level of C3 in LN.

Elevated urinary protein is an important diagnostic indicator of LN. Previous research found that the increased level of complement in the urine of patients with LN was related to the overall increased level of urinary protein [36]. We found that complement components C3aR and C5aR1 were significantly upregulated in the kidneys of LN patients and NZB/W mice. In fact, there is evidence that C3a and C5a are powerful chemoattractants that guide neutrophils, monocytes, and macrophages to the site of complement activation, and thus promote inflammation [37]. Studies of the kidney tissues of patients reported upregulation of C3aR and C5aR at the mRNA and protein levels, and that C3aR or C5aR antagonists reduced the symptoms of LN [26, 27, 38].

We found that the levels of C1q and C4 were positively correlated with the blood level of C3. As an upstream factor, C1q activates C3 with the help of C4 in the classical complement pathway. However, our GO analysis showed that upregulated SERPING1 and A2M were significantly enriched in the GO term of negative regulation of complement activation lectin pathway in the kidneys of LN patients and NZB/W mice. This result suggests that the downstream signaling of C1q is significantly inhibited by SERPING1 and A2M in the kidneys of these patients. C1q mediates a variety of immunoregulatory functions that are considered important in the prevention of autoimmunity, such as enhancement of phagocytosis and regulation of cytokine production by antigen-presenting cells [39]. A deficiency of C1q strongly predisposes individuals to SLE [40], which is thought to be related to the role of C1q in the removal of apoptotic cells [41] and the clearance of immune complex [42]. The lupus autoantigen on the surface of apoptotic cells appears to stimulate an inappropriate immune response in SLE [43–45]. Decreased serum levels of C1q protein and increased titers of C1q antibodies may be involved in the pathogenesis of SLE, especially LN [46]. The prognosis of LN patients is poor when there is no C1q deposition in the kidneys [47]. Taken together, these results support the conclusions that C3 is a key pathogenic factor in the kidneys of LN patients and that the downstream signaling of C1 is inhibited in the kidneys of these patients.

We searched for a factor other than C1, that could potentially regulate complement C3 in LN patients. The results indicated that LN patients had a decreased plasma level of TGFβ1, but the urinal level was increased. The urinary level of TGFβ1 also had a positive correlation with the level of total urinary protein. Our analysis of the peripheral blood and kidney tissues of patients with LN indicated a correlation in the levels of TGFβ1 and C3 at the mRNA and protein levels. In addition, our primary cells-cultures indicated that TGFβ1 promoted the expression of C3 and that a TGFβ1 antagonist (SB431542) decreased the levels of C3 and C3aR. In agreement, other researchers found that TGFβ1 modulated C3 in cultured human monocytes [48] and another research group observed that the serum TGFβ1 level had positive correlations with eGFR, C3, and C4 in SLE patients [49]. Therefore, the data presented here support the interpretation that TGFβ1 modulates C3, and they both function in the pathogenesis of LN.

Many previous studies examined the effect of TGFβ1 inhibition as treatment for various diseases. For example, clinical trials of systemic sclerosis examined the effect of TGFβ inhibitors [50]. Some researchers believe that anti-TGFβ therapy is most likely to be effective during the early stages of systemic sclerosis, before the development of irreversible tissue fibrosis [51]. In addition, the pathogenic effect of TGFβ in systemic sclerosis appears to vary among patients [51]. TGFβ expression is generally greater during early-stage disease active systemic sclerosis, but is weak or undetectable in patients with established skin fibrosis. This may explain the negligible effects of TGFβ inhibitors in some patients with systemic sclerosis. To examine the possible therapeutic benefits of a TGFβ1 inhibitor in LN, we treated NZB/W mice with a TGFβ1 antagonist (SB431542). This inhibitor markedly decreased the renal expression of C3, C3aR, and other complement components in vivo. It is therefore possible that the administration of a TGFβ1 inhibitor to patients with LN may reduce the deposition of immune complexes, the inflammatory response, and renal fibrosis. Some evidence indicates that SB431542 may be a novel treatment for renal fibrosis [52]. Thus, SB431542 or another suitable TGFβ1 inhibitor may help to inhibit the deposition of complement components in the kidneys and provide effective treatment of LN. The usage of this approach for the treatment of LN requires further research.

Our research has several limitations. First, our studies of NZB/W mouse kidney tissues indicated increased C3 deposition as the disease progressed. However, because renal puncture is an invasive operation, we cannot track the deposition of C3 in human kidneys during the progression of LN. Thus, it is uncertain whether C3 gradually increases in the kidneys of LN patients as the disease progresses. Second, the source of the complement components we measured in the kidneys is not completely clear. Masanobu Miyazaki et al. [53] reported that C3 mRNA was observed in mesangial cells, glomerular epithelial cells, and Bowman's capsule in LN, which suggest that locally synthesized complement may be involved in tissue injury. In addition, it is found that C1q produced by macrophages are closely related to the occurrence of LN [54]. Therefore, we speculate that complement components measured at the mRNA level may be from renal intrinsic cells or infiltrating inflammatory cells, and the complement components measured at the protein level may be from renal intrinsic cells, infiltrating inflammatory cells, or peripheral blood. Third, although we found a correlation of C3 and TGFβ1 expression, we did not establish a causal relationship. However, previous studies found that the combination of C3a and C3aR upregulated the expression of TGFβ1, and thereby promoted the progression of pulmonary fibrosis [55]. Nonetheless, further evidence is needed to confirm that C3 increases TGFβ1 expression in patients with LN.

## Conclusions

There were significantly greater levels of C3 and other complement pathway-related factors in the kidneys of patients with LN and NZB/W mice. C3 may participate in the pathogenesis of LN and contribute to albuminuria. TGFβ1 promotes the synthesis of C3, and TGFβ1 inhibition may block the progression of LN by inhibiting the synthesis of C3 and other complement components.

## Supplementary Information

**Additional file 1: Supplementary Table S1.**. Clinical data of study subjects.

**Additional file 2: Supplementary Figure S1.**. Volcano plot of genes differentially expressed in NZB/W mice (top) and patients with LN (bottom). Each point represents a gene that was detectable in both groups. Red point: upregulated genes; green point: downregulated genes.

**Additional file 3: Supplementary Figure S2.**. Enrichment and *p* values of upregulated KEGG pathways in the kidneys of LN patients. Dot size represents the number of genes enriched in the pathway, dot color represents the significance of each differential expression pathway, and dot position represents the enrichment degree of the pathway.

**Additional file 4: Supplementary Table S2**. Detailed information of LN related pathways.

**Additional file 5: Supplementary Figure S3**. Levels of C1q and C4 in plasma of SLE patients. (A) C1q in SLE patients without LN (N = 38) and patients with LN (N = 28). * p < 0.05. (B) C4 in SLE patients without LN (N = 76) and patients with LN (N = 83). (C) Correlation of the protein levels of C1q and C3 in plasma (N = 64). (D) Correlation of the protein levels of C4 and C3 in plasma (N = 145).

**Additional file 6: Supplementary Figure S4**. Immunohistochemical analysis of C3 in glomeruli (GLO) or renal tubules (TUB) of different classes of LN.

## Data Availability

The datasets used and/or analyzed during the current study are available from the corresponding author on reasonable request.

## Abbreviations

ACR: American College of Rheumatology
C1: Complement C1
C3: Complement C3
C3aR1: Complement C3a receptor 1
C5aR1: Complement C5a receptor 1
CR3: Complement component 3 receptor 3
CR4: Complement component 3 receptor 4
ECM: Extracellular matrix
ELISA: Enzyme-linked immunosorbent assay
FBS: Fetal bovine serum
FPR: Formyl peptide receptor
GO: Gene Ontology
KEGG: Kyoto Encyclopedia of Genes and Genomes
LN: Lupus nephritis
PBMC: Peripheral blood mononuclear cell
PDGF-B: Platelet-derived growth factor B
qPCR: Real-time quantitative PCR
SLE: Systemic lupus erythematosus
SLEDAI: SLE Disease Activity Index
TGFβ: Transforming growth factor-β
TLR9: Toll-like receptor 9
