# Supplementary material for: Microarray-based analysis of renal complement components reveals a therapeutic target for lupus nephritis
Source: Arthritis Res Ther. 2021 Aug 25;23:223. doi: 10.1186/s13075-021-02605-9 (PMC8385907; doi:10.1186/s13075-021-02605-9)
Supplement: Supplementary file 1 — Additional file 1: Supplementary Table S1.. Clinical data of study subjects. [file 13075_2021_2605_MOESM1_ESM.pdf]

Supplementary Table S1. Clinical data of the study subjects

	Ctrl (n = 47)	SLE without LN (n = 69)	LN (n = 76)	p value Ctrl vs. SLE without LN	Ctrl vs. LN	SLE without LN vs. LN
**Gender (female/male)**	42/5	65/4	72/4	0.4821	0.3006	0.8891
**Median age (range), years**	38 (23 - 72)	36 (15 - 67)	40 (15 - 70)	0.1102	0.4051	0.3627
**SLEDAI**	-	9.71 ± 4.27	20.04 ± 6.23	-	-	<0.0001***
**Proteinuria, g/24 hour**	-	0.14 ± 0.04	2.24 ± 2.91	-	-	<0.0001***
**Anti-dsDNA antibody-positive, %**	-	74	88	-	-	0.0334*
**Complement C3, g/L**	-	0.55 ± 0.22	0.41 ± 0.23	-	-	<0.0001***

Quantitative data with a normal distribution are presented as mean ± SD. Quantitative data with a non-normal distribution are presented as median (IQR).
p value: * $p < 0.05$, ** $p < 0.01$, *** $p < 0.001$.
