# Supplementary material for: Microarray-based analysis of renal complement components reveals a therapeutic target for lupus nephritis
Source: Arthritis Res Ther. 2021 Aug 25;23:223. doi: 10.1186/s13075-021-02605-9 (PMC8385907; doi:10.1186/s13075-021-02605-9)
Supplement: Supplementary file 2 — Additional file 2: Supplementary Figure S1.. Volcano plot of genes differentially expressed in NZB/W mice (top) and patients with LN (bottom). Each point represents a gene that was detectable in both groups. Red point: upregulated genes; green point: downregulated genes. [file 13075_2021_2605_MOESM2_ESM.pdf]

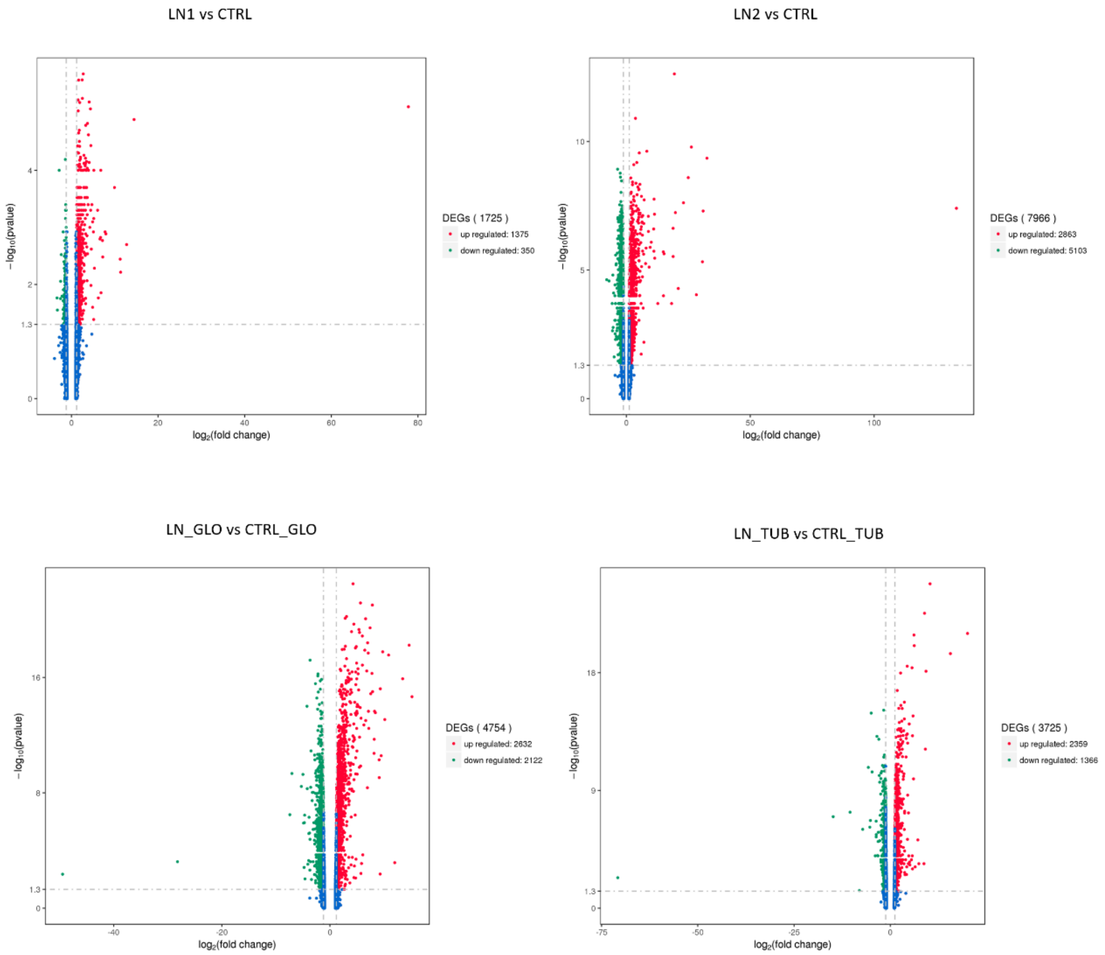

Additional file 2: Supplementary Figure S1. Volcano plot of genes differentially expressed in NZB/W mice (top) and patients with LN (bottom). Each point represents a gene that was detectable in both groups. Red point: up-regulated genes; green point: down-regulated genes.
