# Supplementary material for: Microarray-based analysis of renal complement components reveals a therapeutic target for lupus nephritis
Source: Arthritis Res Ther. 2021 Aug 25;23:223. doi: 10.1186/s13075-021-02605-9 (PMC8385907; doi:10.1186/s13075-021-02605-9)
Supplement: Supplementary file 3 — Additional file 3: Supplementary Figure S2.. Enrichment and p values of upregulated KEGG pathways in the kidneys of LN patients. Dot size represents the number of genes enriched in the pathway, dot color represents the significance of each differential expression pathway, and dot position represents the enrichment degree of the pathway. [file 13075_2021_2605_MOESM3_ESM.pdf]

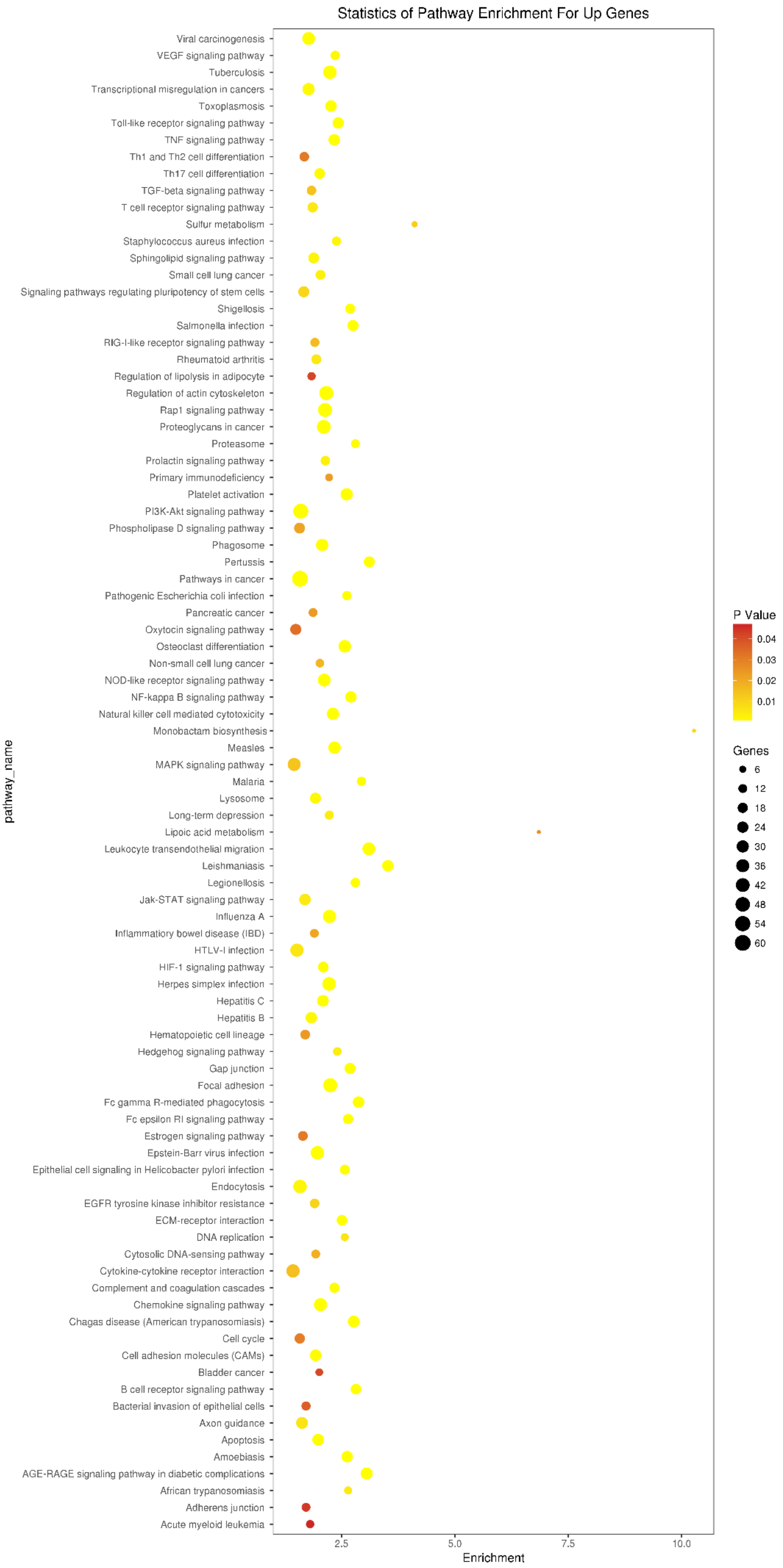
Statistics of Pathway Enrichment For Up Genes
Viral carcinogenesis
VEGF signaling pathway
Tuberculosis
Transcriptional misregulation in cancers
Toxoplasmosis
Toll-like receptor signaling pathway
TNF signaling pathway
Th1 and Th2 cell differentiation
Th17 cell differentiation
TGF-beta signaling pathway
T cell receptor signaling pathway
Sulfur metabolism
Staphylococcus aureus infection
Sphingolipid signaling pathway
Small cell lung cancer
Signaling pathways regulating pluripotency of stem cells
Shigellosis
Salmonella infection
RIG-I-like receptor signaling pathway
Rheumatoid arthritis
Regulation of lipolysis in adipocyte
Regulation of actin cytoskeleton
Rap1 signaling pathway
Proteoglycans in cancer
Proteasome
Prolactin signaling pathway
Primary immunodeficiency
Platelet activation
PI3K-Akt signaling pathway
Phospholipase D signaling pathway
Phagosome
Pertussis
Pathways in cancer
Pathogenic Escherichia coli infection
Pancreatic cancer
Oxytocin signaling pathway
Osteoclast differentiation
Non-small cell lung cancer
NOD-like receptor signaling pathway
NF-kappa B signaling pathway
Natural killer cell mediated cytotoxicity
Monobactam biosynthesis
Measles
MAPK signaling pathway
Malaria
Lysosome
Long-term depression
Lipoic acid metabolism
Leukocyte transendothelial migration
Leishmaniasis
Legionellosis
Jak-STAT signaling pathway
Influenza A
Inflammatory bowel disease (IBD)
HTLV-I infection
HIF-1 signaling pathway
Herpes simplex infection
Hepatitis C
Hepatitis B
Hematopoietic cell lineage
Hedgehog signaling pathway
Gap junction
Focal adhesion
Fc gamma R-mediated phagocytosis
Fc epsilon RI signaling pathway
Estrogen signaling pathway
Epstein-Barr virus infection
Epithelial cell signaling in Helicobacter pylori infection
Endocytosis
EGFR tyrosine kinase inhibitor resistance
ECM-receptor interaction
DNA replication
Cytosolic DNA-sensing pathway
Cytokine-cytokine receptor interaction
Complement and coagulation cascades
Chemokine signaling pathway
Chagas disease (American trypanosomiasis)
Cell cycle
Cell adhesion molecules (CAMs)
Bladder cancer
B cell receptor signaling pathway
Bacterial invasion of epithelial cells
Axon guidance
Apoptosis
Amoebiasis
AGE-RAGE signaling pathway in diabetic complications
African trypanosomiasis
Adherens junction
Acute myeloid leukemia
pathway_name
2.5
5.0
7.5
10.0
Enrichment
P Value
0.04
0.03
0.02
0.01
Genes
6
12
18
24
30
36
42
48
54
60

Additional file 3: Supplementary Figure S2. Enrichment and p-values of up-regulated KEGG pathways in the kidneys of LN patients. Dot size represents the number of genes enriched in the pathway, dot color represents the significance of each differential expression pathway, and dot position represents the enrichment degree of the pathway.
