# Supplementary material for: Microarray-based analysis of renal complement components reveals a therapeutic target for lupus nephritis
Source: Arthritis Res Ther. 2021 Aug 25;23:223. doi: 10.1186/s13075-021-02605-9 (PMC8385907; doi:10.1186/s13075-021-02605-9)
Supplement: Supplementary file 4 — Additional file 4: Supplementary Table S2. Detailed information of LN related pathways. [file 13075_2021_2605_MOESM4_ESM.pdf]

Supplementary Table S2. Description of LN related pathways

Human—LN related differential pathways

pathway_id	pathway_name	population_ mapped_id	study_mapped _id	enrichment	p_value	FDR
hsa04064	NF-kappa B signaling pathway	95	25	2.703451374	2.51797E-06	4.57957E-05
hsa04662	B cell receptor signaling pathway	73	20	2.814552115	1.32198E-05	0.00016726
hsa04620	Toll-like receptor signaling pathway	106	25	2.422904533	2.04284E-05	0.000237786
hsa04610	Complement and coagulation cascades	79	18	2.340709797	0.000455434	0.003313284
hsa04660	T cell receptor signaling pathway	105	19	1.858944659	0.005577142	0.026605711
hsa03030	DNA replication	36	9	2.568278805	0.006311228	0.029151864
hsa04350	TGF-beta signaling pathway	84	15	1.834484861	0.014586711	0.060639042
hsa04110	Cell cycle	124	19	1.574106364	0.030360731	0.109073736
hsa04658	Th1 and Th2 cell differentiation	92	15	1.674964438	0.031300213	0.109761327

Mouse—LN related differential pathways

pathway_id	pathway_name	population_ mapped_id	study_mapped _id	enrichment	p_value	FDR
mmu04620	Toll-like receptor signaling pathway	101	25	3.259616945	8.24785E-08	1.66736E-06
mmu04064	NF-kappa B signaling pathway	102	25	3.227659916	1.01595E-07	1.66736E-06
mmu04662	B cell receptor signaling pathway	74	20	3.559149313	3.55772E-07	4.13585E-06
mmu04610	Complement and coagulation cascades	87	18	2.724590164	7.35911E-05	0.000477486
mmu04660	T cell receptor signaling pathway	105	20	2.508352849	0.000101741	0.000603954
mmu03030	DNA replication	35	9	3.386276347	0.000929161	0.004181225
mmu04110	Cell cycle	124	19	2.017808038	0.002424373	0.009140541
mmu04658	Th1 and Th2 cell differentiation	87	14	2.119125683	0.005564064	0.018439656
mmu04350	TGF-beta signaling pathway	85	12	1.859132112	0.026190335	0.070942753
