# Supplementary material for: Microarray-based analysis of renal complement components reveals a therapeutic target for lupus nephritis
Source: Arthritis Res Ther. 2021 Aug 25;23:223. doi: 10.1186/s13075-021-02605-9 (PMC8385907; doi:10.1186/s13075-021-02605-9)
Supplement: Supplementary file 5 — Additional file 5: Supplementary Figure S3. Levels of C1q and C4 in plasma of SLE patients. (A) C1q in SLE patients without LN (N = 38) and patients with LN (N = 28). * p < 0.05. (B) C4 in SLE patients without LN (N = 76) and patients with LN (N = 83). (C) Correlation of the protein levels of C1q and C3 in plasma (N = 64). (D) Correlation of the protein levels of C4 and C3 in plasma (N = 145). [file 13075_2021_2605_MOESM5_ESM.pdf]

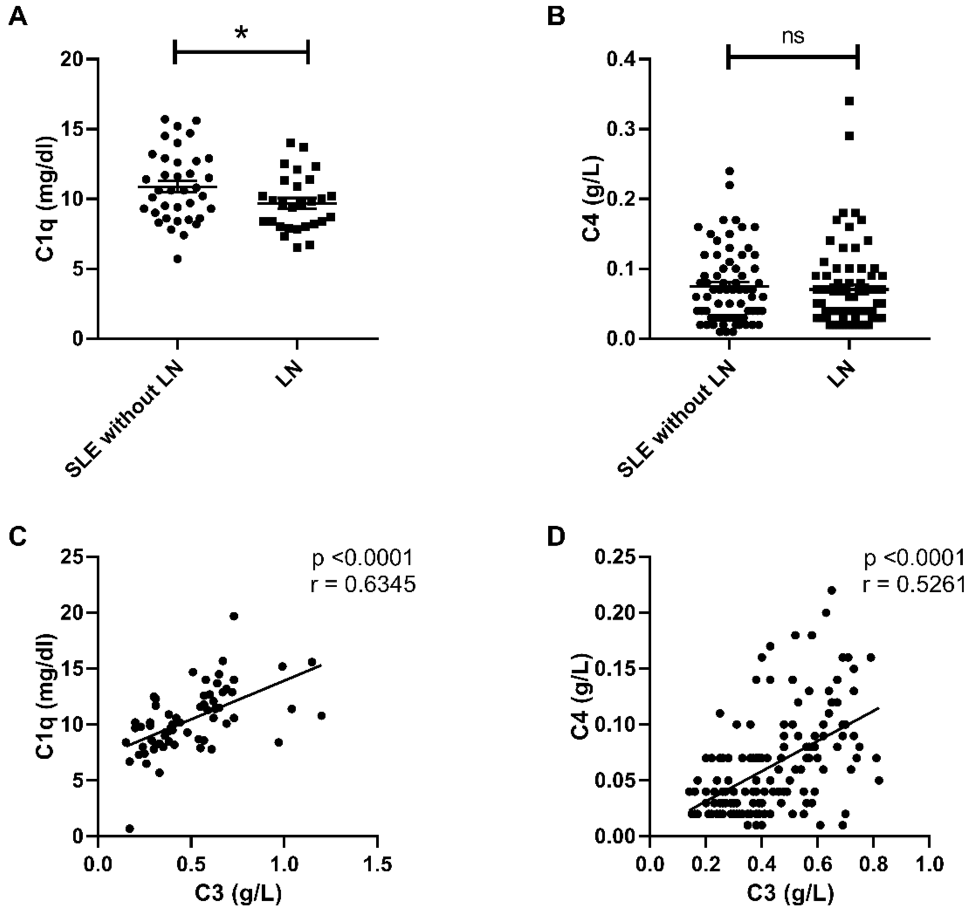

Additional file 5: Supplementary Figure S3. Levels of C1q and C4 in plasma of SLE patients. (A) C1q in SLE patients without LN (N = 38) and patients with LN (N = 28). * $p < 0.05$. (B) C4 in SLE patients without LN (N = 76) and patients with LN (N = 83). (C) Correlation of the protein levels of C1q and C3 in plasma (N = 64). (D) Correlation of the protein levels of C4 and C3 in plasma (N = 145).
