# Supplementary material for: Microarray-based analysis of renal complement components reveals a therapeutic target for lupus nephritis
Source: Arthritis Res Ther. 2021 Aug 25;23:223. doi: 10.1186/s13075-021-02605-9 (PMC8385907; doi:10.1186/s13075-021-02605-9)
Supplement: Supplementary file 6 — Additional file 6: Supplementary Figure S4. Immunohistochemical analysis of C3 in glomeruli (GLO) or renal tubules (TUB) of different classes of LN. [file 13075_2021_2605_MOESM6_ESM.pdf]

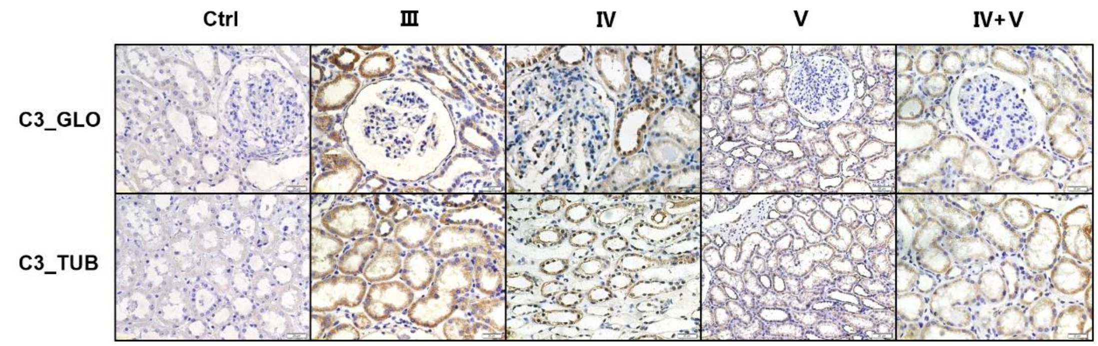

Additional file 6: Supplementary Figure S4. Immunohistochemical analysis of C3 in glomeruli (GLO) or renal tubules (TUB) of different classes of LN.
